# In Silico Evaluation of Quercetin Methylated Derivatives on the Interaction with Secretory Phospholipases A2 from *Crotalus durissus terrificus* and *Bothrops jararacussu*

**DOI:** 10.3390/ph16040597

**Published:** 2023-04-15

**Authors:** Mariana Novo Belchor, Caroline Ramos da Cruz Costa, Airam Roggero, Laila L. F. Moraes, Ricardo Samelo, Isabelly Annunciato, Marcos Antonio de Oliveira, Sergio F. Sousa, Marcos Hikari Toyama

**Affiliations:** 1 Center of Natural and Human Sciences, Federal University of ABC (UFABC), Santo André 09210-580, SP, Brazil; belchor.novo@ufabc.edu.br (M.N.B.);; 2 Biosciences Institute of Paulista Coast Campus (IB/CLP), University of São Paulo State (UNESP), São Vicente 11330-900, SP, Brazil; 3 Unit of Applied Biomolecular Sciences (UCIBIO), REQUIMTE-BioSIM-Medicine Faculty, Porto University, 4050-345 Porto, Portugal

**Keywords:** natural compounds, inflammation, toxins, snake venoms, molecular docking

## Abstract

Quercetin derivatives have already shown their anti-inflammatory potential, inhibiting essential enzymes involved in this process. Among diverse pro-inflammatory toxins from snake venoms, phospholipase A2 is one of the most abundant in some species, such as *Crotalus durissus terrificus* and *Bothrops jararacussu* from the Viperidae family. These enzymes can induce the inflammatory process through hydrolysis at the sn-2 position of glycerophospholipids. Hence, elucidating the main residues involved in the biological effects of these macromolecules can help to identify potential compounds with inhibitory activity. In silico tools were used in this study to evaluate the potential of quercetin methylated derivatives in the inhibition of bothropstoxin I (BthTX-I) and II (BthTX-II) from *Bothrops jararacussu* and phospholipase A2 from *Crotalus durissus terrificus*. The use of a transitional analogous and two classical inhibitors of phospholipase A2 guided this work to find the role of residues involved in the phospholipid anchoring and the subsequent development of the inflammatory process. First, main cavities were studied, revealing the best regions to be inhibited by a compound. Focusing on these regions, molecular docking assays were made to show main interactions between each compound. Results reveal that analogue and inhibitors, Varespladib (Var) and p-bromophenacyl bromide (BPB), guided quercetins derivatives analysis, revealing that Leu2, Phe5, Tyr28, glycine in the calcium-binding loop, His48, Asp49 of BthTX-II and Cdtspla2 were the main residues to be inhibited. 3MQ exhibited great interaction with the active site, similar to Var results, while Q anchored better in the BthTX-II active site. However, strong interactions in the C-terminal region, highlighting His120, seem to be crucial to decreasing contacts with phospholipid and BthTX-II. Hence, quercetin derivatives anchor differently with each toxin and further in vitro and in vivo studies are essential to elucidate these data.

## 1. Introduction

Flavonoids, compounds that are secondary metabolites from plants, have a nucleus which consists of A, B and C rings. A series of modification reactions, such as hydroxylation, glycosylation, prenylation, and methylation, can enhance multiple physiological functions corresponding to both their structural diversity and tissue specificities. The *O*-methylation of aglycone flavonoids, such as Quercetin (Q), results in the reduction of the molecular activity of a hydroxyl fraction and the consequent increase in lipophilicity, which modifies its intracellular compartmentalization. Furthermore, *O*-methylation provides a branch point in the biosynthesis of several metabolic pathways, including production of modified flavonoids with increased antimicrobial properties [1]. Considered a post-modification product, these derivatives are formed through the methyl group fixation with oxygen at the flavonoid hydroxyl moiety. Due to the diverse hydroxyl groups in the flavonoid core, flavonoids’ methylation positions are diverse and provide multiple health benefits, such as increased bioavailability compared to flavonoid precursors [2]. Therefore, these methylated flavonoids are potential candidates for use as anti-inflammatories by decreasing the enzymatic activity of enzymes such as cyclooxygenases (COX), pro-inflammatory interleukins, reactive oxygen species (ROS) and nitrogen (RNS) production [2,3,4]. Previous analyzes using ChEMBL and SwissTargetPrediction tools reveals that both Rhamnetin (Rhm; 7-*O*-Methylquercetin); 3-*O*-Methylquercetin (3MQ; 3-*O*-Methylquercetin) are compounds with an antioxidant capacity, besides to exhibits anti-inflammatory potential by strongly decreasing the cyclooxygenase (COX) and Lipoxygenase (LOX) activity. Rhamnazin (Rhz; 7,3’-Di-*O*-methylquercetin) exhibits two methylations, which seem to considerably increase the ability to sequester free radicals in cells. Methylated derivatives of Q are also found in some plant species, such as *Coriandrum sativum, Achyrocline satureioides*, and *Rhamnus petiolaris* with Rhm, 3MQ, and Rhz compounds, respectively, which have already shown different responses against BthTX-II activities [5].

Snake venoms consist of a complex mixture of biologically active molecules, and the phospholipase A2 (PLA2) group is one of the most studied toxins. There are two main groups of PLA2 (E.C. 3.1.1.4) in snake venoms: phospholipase A2-like (PLA2-like), such as the Lys49-PLA2 and the classic phospholipase A2, Asp49-PLA2. These proteins belong to the secreted PLA2 (sPLA2), a subgroup found in diverse secretions, body fluids and venom of bees, scorpions, and snakes [6]. Therefore, these macromolecules hydrolyze the sn-2 position of glycerophospholipids, leading to a release of fatty acids, such as arachidonic acid (AA) and lysophospholipids, triggering the inflammatory process [7,8]. Bothropstoxin II (BthTX-II) is an Asp49-PLA2 from *Bothrops jararacussu* (Bj), which belongs to the Viperidae family. This toxin is considered a PLA2-like due to some characteristics, such as the Ca^2+^ binding loop distortion, leading to changes in the C-terminal region. In this way, this protein shows a low phospholipase A2 activity besides to exhibit myotoxic, edematogenic, and hemolytic effects [9,10]. Bothropstoxin I (BthTX-I) is a Lys49-PLA2, which reveals high myotoxic activity with a lack of enzymatic activity. The myotoxic mechanism of these enzymes has already been demonstrated and includes mainly some amino acids from the C-terminal region, which interacts with membrane [8].

*Crotalus durissus terrificus* (Cdt) belongs to the same family and has an essential heterodimeric complex named Crotoxin in its venom, which is a potent β-neurotoxin with a phospholipase A2 activity. This toxin consists of two subunits: a basic pla2 with a weak neurotoxicity, named crotoxin B (CB) or Cdt PLA2, associated with a small acidic, nontoxic and nonenzymatic protein named crotapotin or crotoxin A (CA) [11]. CB presents four isoforms, CBa2, CBb, CBc, and CBd, performing 16 different CA-CB complexes. To date, there are three crystal structures from these isomers: one of them includes CA2 and CBb, the other with CBd (tetramer), and the structure used in this study consists of isoforms CBa2 and CBc (tetramer) [12].

Commercial inhibitors of PLA2, such as p-bromophenacyl bromide (BPB) and Varespladib (Var), have already shown their potential against PLA2 from diverse snake venoms. The classical inhibitor BPB has been used since 1970 to inhibit the catalytic PLA2 once it binds specifically with the His48 residue. However, this compound has already been shown to inhibit myotoxic activity of PrTX-I, a Lys49-PLA2 from *Bothrops pirajai*, through a covalent binding to His48, leading to a Ca^2+^-binding loop distortion and then, a C-terminus rearrangement [13]. Var (LY315920) is a synthetic molecule which was clinically tested to block the inflammatory cascade initiated by secreted PLA2. There are several studies that reveal the efficacy of this compound in the treatment of phospholipase A2-rich snake venoms [14]. In snake venoms, this compound has already been shown to inhibit the cytotoxic e myotoxic effect of MjTX-II from *Bothrops moojeni* through the physical blockage of its allosteric activation [15]. In addition to this, a synergic effect of this compound with the antivenom was observed to decrease neuromuscular blockage induced by crotamine, highlighting the broad-spectrum effect of this drug [16].

Due to PLA2 role in the inflammatory process, the abundance of this toxin in the Cdt and Bj venoms, and the fact that these proteins show similar structures with sPLA2 from mammals [12], it is essential to find new inhibitors of this enzyme. Hence, in this study, we performed some steps to better understand how these compounds could interact with these three phospholipases A2. First, we aim to elucidate the main protein’s regions to anchor with an inhibitor using CavityPlus. Afterwards, using the phosphonate transition-state analogue from 1POB crystal structure, named L-1-0-octyl-2-heptylphos-phonyl-sn-glycero-3-phosphoethanolamine (Analogue) [17], we focus on the elucidation of the main residues that interact with each toxin. Moreover, two commercial inhibitors were used for comparison purposes to identify the main residues involved in the anchoring with all toxins. Finally, we intend to compare the compounds’ interactions with phospholipase A2 from *Bothrops jararacussu* and *Crotalus durissus terrificus* with the analogue and the inhibitors. Thus, we analyze if compounds anchor equally with all toxins, and if not, we try to elucidate main differences and the reasons for that.

## 2. Results

### 2.1. Properties of Compounds

Firstly, compounds were evaluated concerning physical–chemical features to better understand their biological activity. Data obtained from SwissADME, Mollinspiration and ChEMBL support these results. Q reveals a molecular weight (MW) of 302.24 g/mol, Rhm and 3MQ show the same value, and Rhz, with two methylations, exhibits a higher MW. The topological polar surface area (TPSA) is higher in Q, the same in Rhm and 3MQ, and Rhz exhibits the smallest value (Table 1). A classical descriptor evaluated lipophilicity: the partition coefficient between *n*-octanol and water, which is essential to analyzing physicochemical properties for pharmacokinetics drug discovery [18].

**Figure 1:**
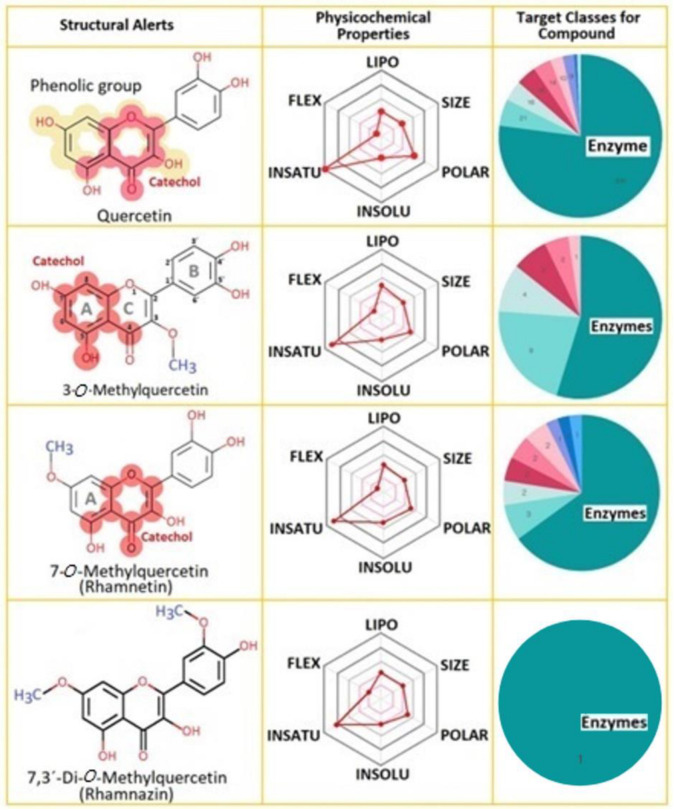
Structural alerts, physicochemical properties of possible targets of Q, Rhm, 3MQ and Rhz. Physicochemical properties in the second column are abbreviated and mean: Lipo: lipophilicity, Flex: Flexibility, Insatu: Insaturations, Insolu: insolubility, Polar: Polarity.

Each compound shows specific alerts related to chemical structures which reveals a high tendency to interact with a great number of molecules and macromolecules. Moreover, the physicochemical properties are exhibited in the second column, revealing specific differences between them, such as a higher amount of unsaturation in Q than the others, besides being more polar. In general, these compounds show that enzymes are their main target (Figure 1).

### 2.2. Molecular Docking

#### 2.2.1. Cavity Analysis

**Figure 2:**
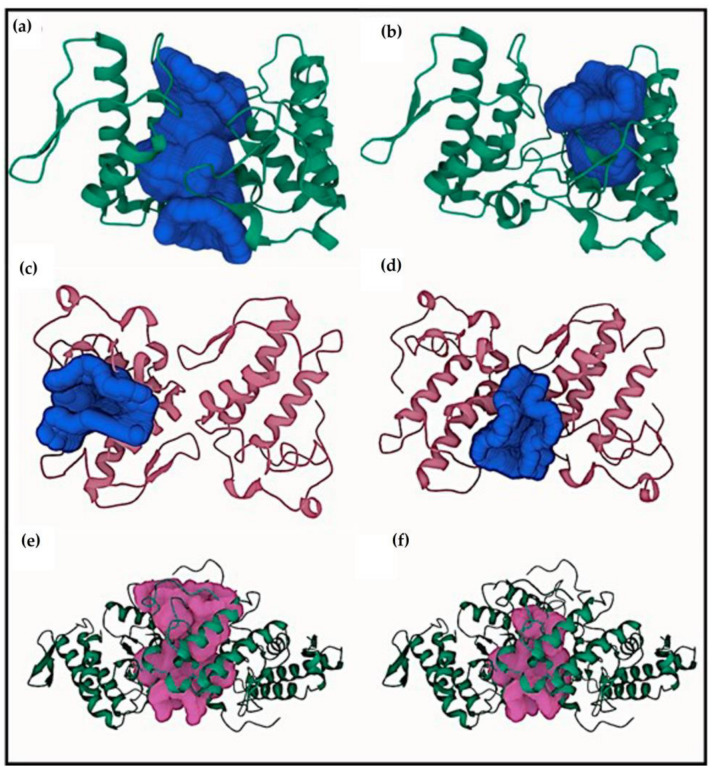
Two best cavities of each protein according to the highest druggability values. (**a**) First cavity observed in BthTX-I between chain A and B and the second region exhibited in the chain A hydrophobic channel (**b**). In (**c**), the highest value of druggability shows cavity in the chain B active site, and the second, in the interface between chain A and B (**d**). In (**e**), CdtsPLA2 reveal a huge area in the interface, and a small one, also in the interface (**f**).

The cavities of each toxin were evaluated using the CavityPlus web server to detect potential binding sites on the protein surface. Figure 2 shows two cavities of each protein with the first and the second highest values of druggability. Figure 2a exhibits the first cavity of BthTX-I between chains A and B and the second (Figure 2b) in the hydrophobic channel, which includes residues such as Gly30, Tyr22, Lys7, Tyr52, Lys69, Ala19, Cys45, Lys69, Lys49, His48, and Ile9. Figure 2 reveals the binding region in the active site of BthTX-II (Figure 2c) and in the interface between chains A and B (Figure 2d). On the other hand, the highest values of druggability found in CdtsPLA2 were in the tetramer interface, including the active sites of the protein (Figure 2e,f).

#### 2.2.2. Docking Analysis with a Transitional Analogous

**Figure 3:**
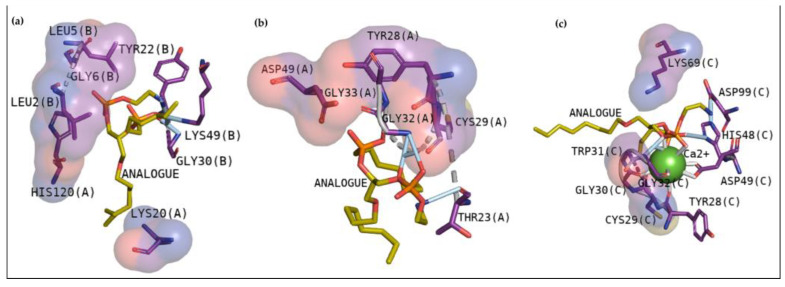
Interactions between BthTX-I (**a**), BthTX-II (**b**) and CdtsPLA2 (**c**) with a phosphonate transition-state analogue.

Figure 3a shows that the molecule establishes hydrogen bonds with Gly30, Lys49, and Tyr22 of chain B of BthTX-I. Hydrophobic contacts with Leu2, Leu5, and Gly6 of the same chain are observed, besides essential contacts with Lys20 and His120 of chain A. BthTX-II shows hydrogen bonds with Gly32–33 and Thr23, despite presenting hydrophobic contacts with Asp49, Tyr28 and Cys29 (Figure 3b). Glycine is also essential in CdtsPLA2 in its interaction with the molecule, once hydrogen bonds were observed with Gly32 with diverse interactions with calcium ion, besides the hydrogen bond with His48 (Figure 3c). Hydrophobic contacts with Cys29, Gly30, Trp31 and Lys69 are also observed. Hence, these are the main residues that must be inhibited to prevent phospholipid hydrolysis and its entrance.

#### 2.2.3. Classical Inhibitors BPB and Var

**Figure 4:**
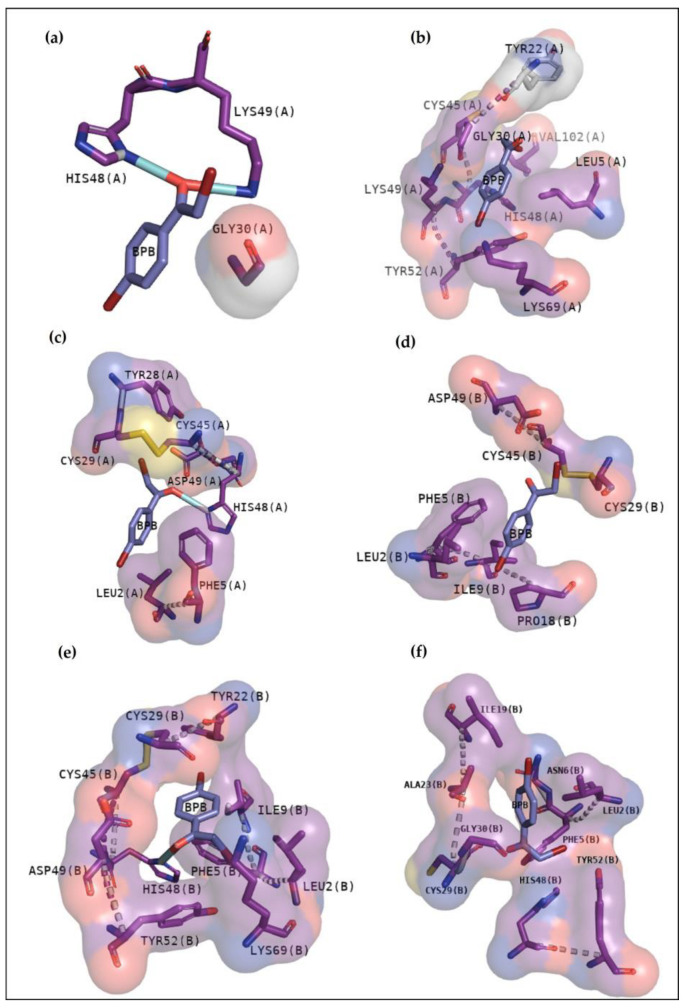
BPB docking results with BthTX-I, BthTX-II and CdtsPLA2. BthTX-I exhibits in the first cluster hydrogen bonds with His48 and Lys49, besides to exhibit hydrophobic contacts with Gly30 (**a**), while second cluster shows hydrophobic contacts with N-terminal residues, Gly30, His48, Lys49, Tyr52 and Lys69 (**b**). Hydrogen bond with His48 is also observed in BthTX-II in the first cluster, besides to show hydrophobic contacts with N-terminal region (**c**), and hydrophobic contacts in the second cluster (**d**). Similarly, BPB with CdtsPLA2 reveal hydrogen bond with His 48 and hydrophobic contacts with essential amino acids (**e**), and (**f**) exhibits just hydrophobic contacts. Purple and red colors around residues represent hydrophobic contacts with the compound.

The compound BPB was used as a guide once it became a classic inhibitor of PLA2 [19]. It mainly shows contacts involved in the catalytic activity of these enzymes or in the channel responsible for phospholipid entrance in BthTX-I. Figure 4 exhibits that all toxins present great interactions with residues essential to the phospholipid fitting (BthTX-I, a and b) or in the active site (BthTX-II (c and d)) and CdtsPLA2 (e and f). Table 2 shows affinity values and rmsd of each docking analysis.

**Figure 5:**
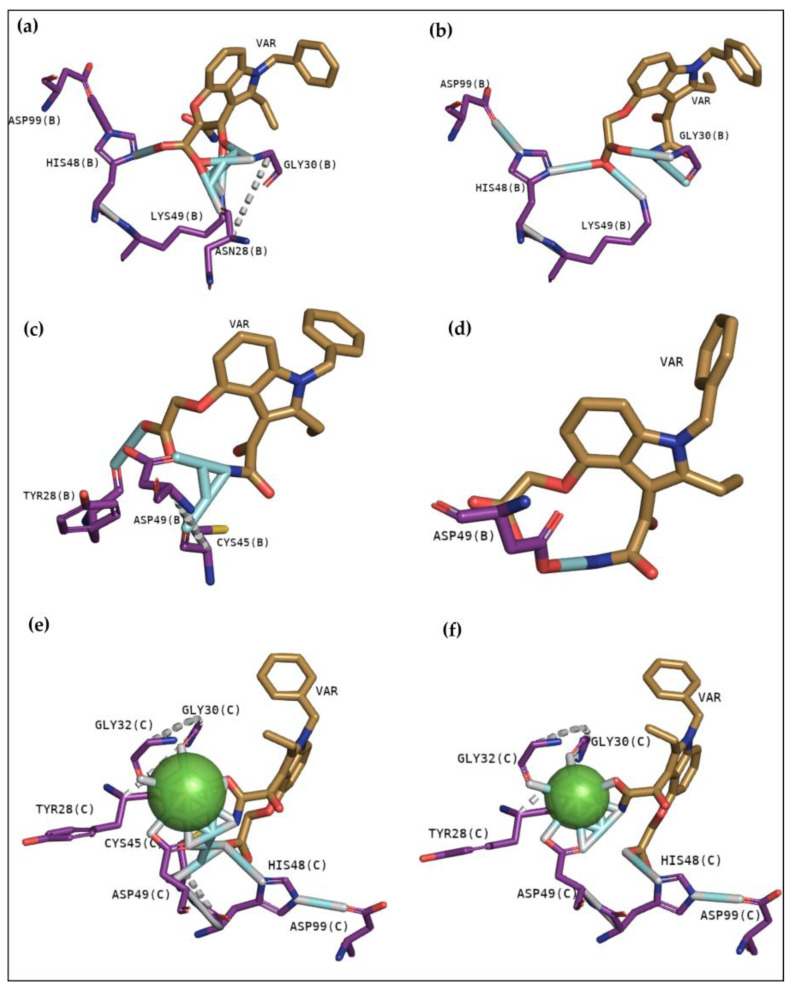
Var docking results with BthTX-I in the first cluster, (**a**) and second cluster (**b**) revealing hydrogen bonds with Gly30, His48 and Lys49. BthTX-II-Var in the first cluster (**c**), with hydrogen bonds with Asp49 and Tyr28, and just with Asp49 in the second cluster (**d**). CdtsPLA2 anchoring with Var, exhibiting strong interactions with the active site and calcium binding loop in the first (**e**) and second (**f**) cluster. Blue cylinders represent hydrogen bonds between residues and the compound.

Results with the inhibitor Var are exhibited in Figure 5. BthTX-I shows hydrogen bonds with Gly30, Asn28, Lys49, and His48 in the first cluster, with a binding free energy of −8.7 kcal/mol (Table 3). Similarly, hydrogen bonds were observed with the same residues, except Ans28 in the second cluster with −8.5 kcal/mol of affinity (Figure 5a,b). Results with BthTX-II revealed less affinity than the other two toxins, and both clusters show −7.1 and −6.7 kcal/mol, referring to clusters 1 and 2, respectively (Figure 5c,d). Residues involved in the anchoring observed in Figure 4c,d were Cys45, Asp49, and Tyr28, also revealing a lower number of hydrogen bonds when compared with BthTX-I and CdtsPLA2. Figure 4e,f present the same residues involved in the CdtsPLA2 interaction with Var, with hydrogen bonds with residues present in the calcium-binding loop, such as Tyr28, Gly30 and 32, besides with Asp49 and His48, residues involved in the protein-catalytic network (Figure 5e,f). In this case, binding free energy revealed −8.3 and −8.2 kcal/mol in clusters 1 and 2, respectively.

#### 2.2.4. BthTX-I

**Figure 6:**
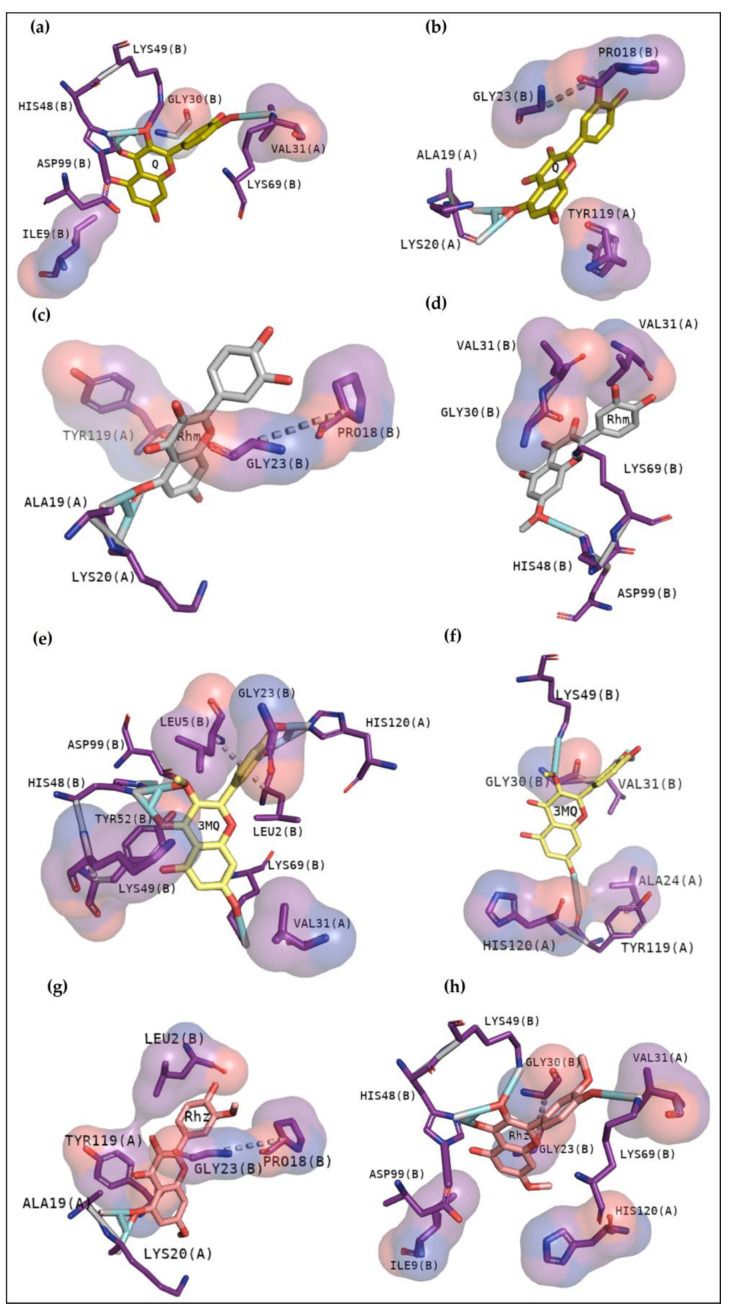
Docking results of BthTX-I with Q in the first cluster (**a**) and the second cluster (**b**), revealing great interactions with His48, Lys49 and Lys69. BthTX-I-Rhm anchoring, emphasizing Lys20, Tyr119 in the first cluster (**c**) and His48, Lys49 and Gly30 in the second (**d**). 3MQ exhibit in the first image (**e**) to anchor strongly with His48 plus Lys69, and in the second with Gly30, Lys49 and the C-terminal region (**f**). Lys20 and Tyr 119 also appears anchoring with Rhz (**g**) and Lys49 and His48 are the strong interactions in the second cluster (**h**). Hydrogen bonds are represented by blue cylinders, and hydrophobic contacts by purple and red colors around the residues.

Docking analysis with BthTX-I reveals that quercetin derivatives can interact with the hydrophobic channel responsible for anchoring with phospholipids. Hydrogen bonds with His48 and Val31, or Ala19 and Lys20, were observed in Figure 6a,b, which corresponds to clusters 1 and 2, respectively, of Q and BthTX-I. Rhm exhibits great interactions between the chains in the first cluster, with hydrogen bonds in Ala19 (A) and Lys20(A), besides the hydrophobic contacts with residues of both protein chains. Figure 6d also shows that Rhm can exhibit hydrogen bonds with residues essential to the phospholipid anchoring, such as His48, Gly30, and Lys69 (Figure 6c,d). Hydrogen bonds with His48, Lys49, Lys69, and His120 were found in the two clusters of 3MQ with the toxin, besides presenting hydrophobic contacts with essential residues from both chains. Similar to Rhm, Rhz exhibits great contact with both chains, revealing hydrogen bonds with Ala19, Lys20, His48, Lys49, and Lys69. Furthermore, hydrophobic contacts with essential amino acids, such as Gly30 and Val31, were observed. Table 4 shows the values of affinity and rmsd from each analysis.

#### 2.2.5. BthTX-II

**Figure 7:**
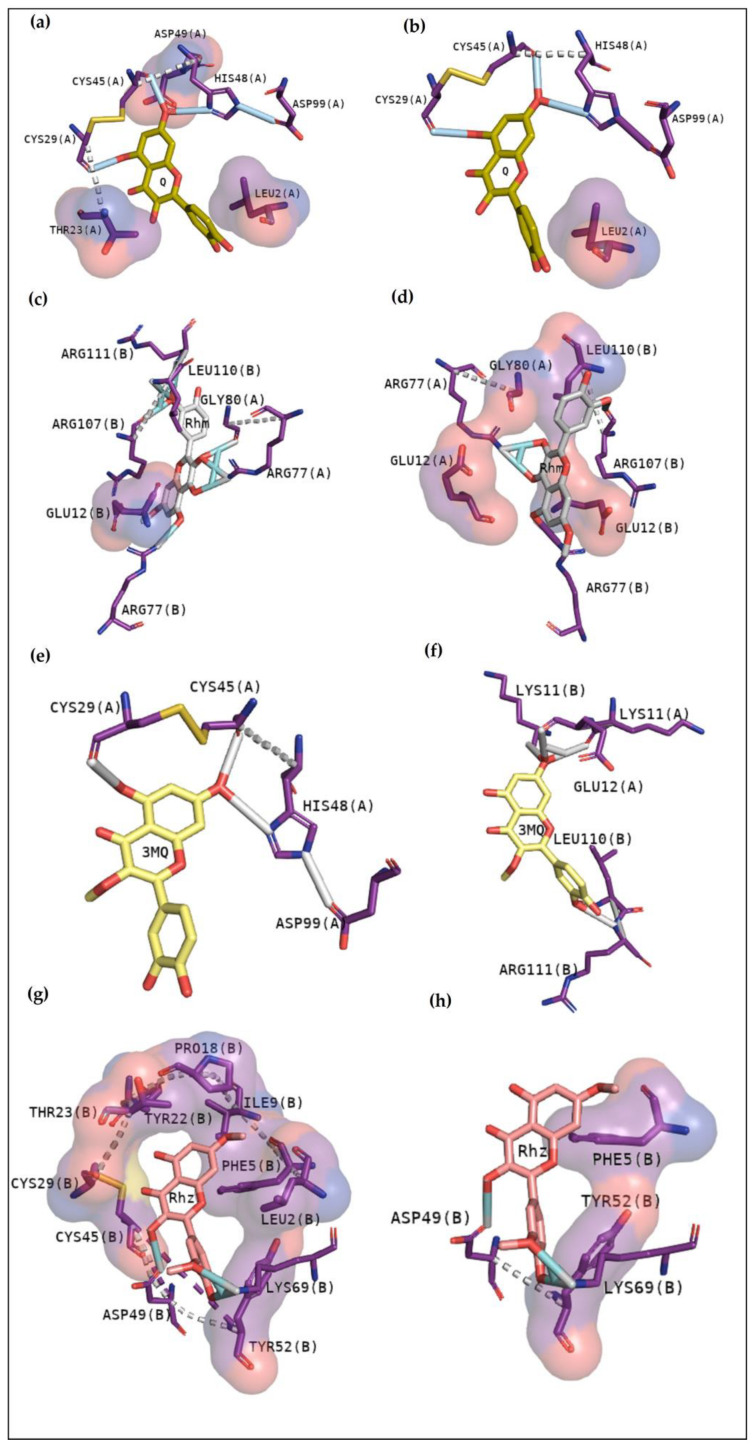
Docking results of BthTX-II with quercetin derivatives. Q reveals to anchor with the active site in the first (**a**) and also in the second cluster (**b**), while Rhm fits in the interface between the chains, emphasizing Gly80, Leu110 and Arg 111 in the first (**c**) and second clusters (**d**). First, 3MQ reveals hydrogen bond with His48 and Cys45 (**e**), and in the second cluster, it was observed contacts with N-terminal and C-terminal region with both chains (**f**). Rhz first shows to anchor with active site and the N-terminal region with mostly hydrophobic contacts (**g**), and in the last cluster, similar residues are shown (**h**).

Cluster 1 of BthTX-II:Q exhibits hydrogen bonds with His48, Cys29, and Cys45 and hydrophobic contacts with Asp49, Thr23, and Leu2 of chain A, with a binding energy of −7.4 kcal/mol(Figure 7a). The second cluster shows hydrogen bonds with Cys29, Cys45, and His48, besides hydrophobic contact with Leu2 (Figure 7b). Rhm was the unique compound which exhibits great interactions in the interface between monomer A and B, showing hydrogen bonds with Arg111(B), Leu110(B), Arg107(B), Gly80(A), Arg77(A), Arg77(B), and Glu12(B) with a binding energy of −7.7 kcal/mol (Figure 7c). The second cluster reveals similar results, with contacts between chains A and B (Figure 7d). Comparable with Q, 3MQ exhibits hydrogen bonds with Cys29, Cys45, and His48. However, the energy affinity was lower, −6.9 kcal/mol, and there were no hydrophobic contacts. Otherwise, the second cluster is between the chains with lower affinity (Figure 7e,f). On the other hand, Rhz shows a hydrogen bond with Asp49 and Lys69, despite presenting hydrophobic interactions with Leu2, Phe5, Ile9, Pro18, Tyr22, Thr23, Cys29, Cys45, and Tyr52, with an energy of −7.3 kcal/mol. Hydrogen bonds with Asp49 and Lys69 were observed in the second cluster; additionally, hydrophobic contacts with Phe5 and Tyr52 were exhibited. Table 5 shows the affinity values and their respective rmsd.

#### 2.2.6. CdtsPLA2 Molecular Docking

**Figure 8:**
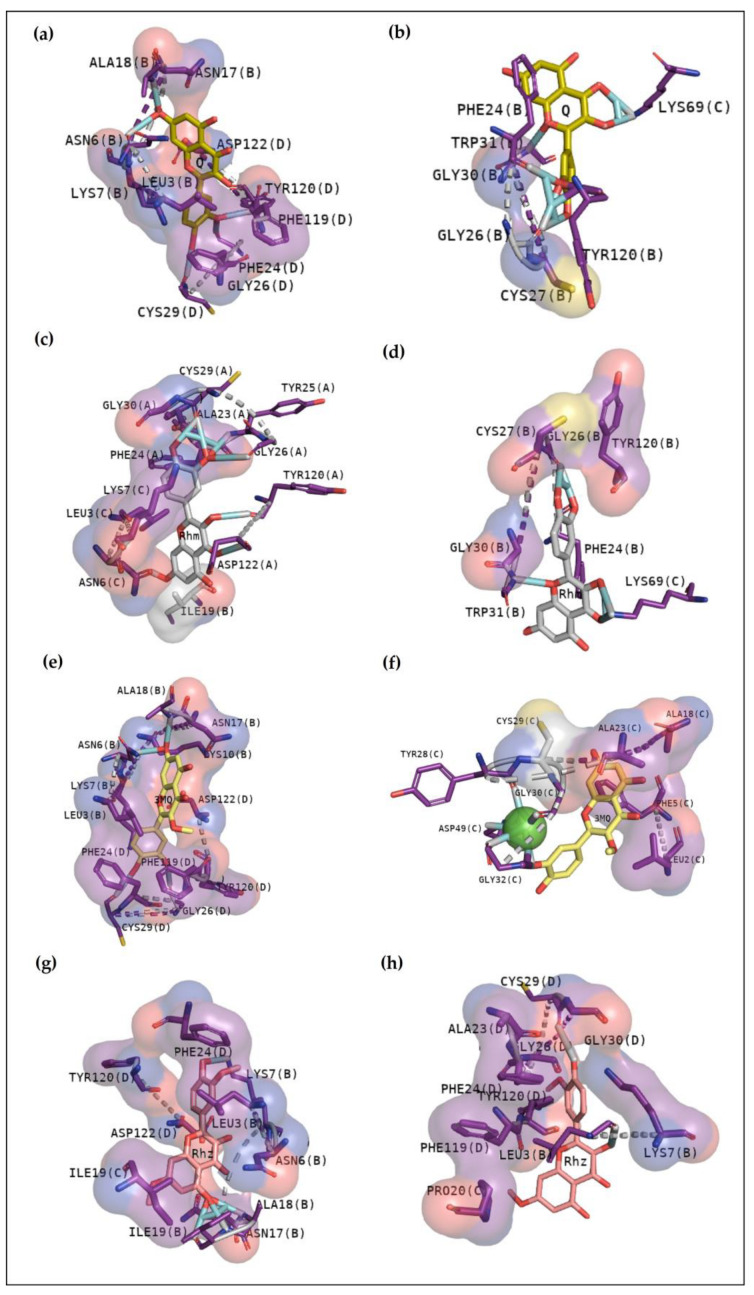
Docking results of CdtsPLA2 with Quercetin derivatives. (**a**) First cluster of Q revealing interactions between chain B and D, and the second, between B and C (**b**). Chain A, B and C were involved in the first Rhm anchoring with the protein (**c**), however, just B and C were observed in the second (**d**). Differently, with a high number of hydrophobic contacts, the first cluster of 3MQ anchors between chains B and D (**e**), and the second one fits in the protein active site (**f**). Similar residues are involved in the Rhz-CdtsPLA2 anchoring, with hydrophobic contacts in the first (**g**) and second cluster (**h**).

Q mostly interacts with the interface of the chains B and D or B, C and D and does not exhibit contact with the active site in the first cluster, which exhibited an affinity of −9.7 kcal/mol, with hydrogen bonds with residues from the N- terminal region and Gly26, Cys29, and Tyr120. Hydrophobic contacts between chains B and D were also observed (Figure 8a). The second cluster presents less affinity (−8.0 kcal/mol) (Table 6) and shows a hydrophobic contact with an amino acid which is fundamental to the catalytic activity: Gly30. In addition to this, it shows hydrogen bonds with Lys69, Trp31, Phe24, Gly26, and Tyr120 (Figure 8b). Rhm showed the first cluster at the interface with chains A and C, exhibiting hydrogen bonds with Cys29, Tyr25, Gly26, Tyr120, and Asp122. Hydrophobic contacts were observed with the N-terminal region and Gly30 (Figure 8c), with an affinity of −9.4kcal/mol. Figure 8d shows hydrogen bonds with Lys69, Trp31, Phe24, and Gly26, and hydrophobic contacts were observed with Gly30, Tyr120, and Cys27. 

3MQ reveals an energy affinity of −9.4 kcal/mol with hydrogen bonds with Asn6, Ala18, Gly26 and Cys29. Diverse hydrophobic contact between chains B and D were observed around the molecule (Figure 8e). The second cluster showed great interactions with CdtsPLA2 active site, with an energy affinity of −8.5 kcal/mol (Table 6). Figure 8f also highlights the hydrogen bonds with Gly32, an essential amino acid in the calcium bind loop. Furthermore, all residues are connected with calcium ions, which is essential to catalytic activity. In addition, hydrophobic contacts with N-terminal amino acids and Cys-29 were observed. The first cluster of Rhz shows interactions between monomers B, C, and D, with a bind free energy of −8.9 kcal/mol. Similarly, with −8.8 kcal/mol, the second cluster exhibits interactions between monomers B, C, and D. Rhz does not show contact with residues directly involved in the catalytic activity of CdtPLA2.

## 3. Discussion

To date, diverse in silico tools help to evaluate molecular structures, mainly to select compounds which exhibit great possibility to become an effective drug [13]. These tools also have an essential role in identifying the interaction of target compounds [20]. The TPSA is an important descriptor to understand the specific regions of the protein-compound’s interactions, and Rhz shows the smallest value. Rhm and 3MQ show a similar value, and Q exhibits the highest polar area, which exceeds the range (20–130 Å^2^) [13]. Therefore, lipophilicity could be another descriptor to support the results obtained in the molecular docking analysis. Diverse natural compounds, such as flavonoids, exhibit an inhibitory potential against sPLA2 activity that seems to be dependent on the 5-hydroxyl group, besides the double bond and the double-bonded oxygen in the oxane ring and the hydroxyl groups at the 3′ and 4′ position [21]. Q is widely found as a secondary metabolite in fruits, vegetables, and flowers. Its structure consists of three rings, consisting of a basic nucleus of a phenyl benzo (γ) pyrone and the side groups usually are hydroxyl, glycosyl, or methoxyl [22].

In recent studies, the search to provide an alternative or complementary treatment to antivenom therapy using synthetic and natural compounds has been the subject of investigation. Var is a synthetic compound known for its inhibitory potential against human-secreted groups IIA PLA2. Due to their high structural homology with PLA2 from snake venoms, studies were made to verify its inhibitory potential [10]. Furthermore, this compound has already been shown to inhibit myotoxins revealing great contacts with Gly30, Lys49 and His48 of MjTX-I and MjTX-II from *Bothrops moojeni* [15]. Additionally, Var also shows to anchor with other myotoxins, such as PrTX-I from *Bothrops pirajai* and BthTX-I from *Bothrops jararacussu* [10]. Herein, results with inhibitor Var reveal that the compound shows higher affinity by BthTX-I from *B. jararacussu*, with great contacts with His48, Lys49, and Gly30, residues also observed in the analogue interactions and in other phospholipases from some *Bothrops* sp venoms [15]. Although BPB interacted with essential amino acids to anchor with the analogue (Figure 3), it exhibits lower values of affinity when compared with Var. This inhibitor is known to decrease the enzymatic activity of phospholipase A2, binding covalently to His48 of PrTX-I [13,19]. This interaction leads to a distortion of the Ca^2+^-binding loop plus a C-terminus rearrangement, decreasing the myotoxic activity of this protein. In this study, BPB also exhibits interactions with His48, Lys49, Leu5, Gly30, and Lys69 of BthTX-I and with the main residues of BthTX-II and CdtsPLA2 active site. In addition, earlier studies reveal that BPB also fits well in the hydrophobic channel with extensive hydrophobic interactions with the surrounding residues, especially Phe5, Cys45, and Gly30 from bovine pancreatic PLA_2_, besides reducing edema induced by PrTX-I in rat and rabbit [22,23]. Moreover, it has already demonstrated the His48 chemical modification of the acid phospholipase A2 from *B. jararacussu* using BPB [24].

Cavity analysis has been used to identify potential binding sites on the protein surface besides ranking them based on ligandability and druggability scores [25]. In this study, this web server helped to confirm and guide the best sites of the protein to focus on molecular docking. To better understand the compounds’ potential to interact with these toxins, it is essential to emphasize the residues involved in the anchoring between these three toxins with the phosphonate transition-state analogue. In Figure 2a, BthTX-I revealed that Lys20, Gly30, Lys49, Tyr22, His120, and some N-terminal residues are involved in anchoring the compound-toxin. BthTX-II exhibited contacts with Tyr28, Gly32,33, and Asp49, and CdtsPLA2 shows great interactions with Gly30, 32, His48, and Asp49. Similarly, Q shows great contact with some of these residues of BthTX-I, such as Lys20, Lys49, Gly30, and His48. Quercetin has already been shown to bind with the dimmer interface and active site of MTX-II, a Lys-49 PLA2 from the *Bothrops brazili* venom [26]. Furthermore, in this study, Q fits well in the active site of BthTX-II, and these data are supported by a previous study in which the compound inhibited the protein enzymatic activity [5]. Q with CdtsPLA2 exhibited similar interactions with BPB, such as Lys69, Gly30, and some residues of the N-terminal region. In addition, Q anchors in the interface between monomers B and D in both analyses, with a great value of affinity, higher than that observed with BthTX-II. This toxin in the solution presents dimeric or tetrameric oligomers [27]. Hence, it may be necessary to use a higher concentration of inhibitor compared to that was used in BthTX-II, once the protein is primarily in a monomeric form in its relaxed state with a fatty acid in its hydrophobic channel [28].

Rhm reveals a different region with high affinity with BthTX-I, the interface between chain A and B, with hydrogen bonds with Lys20 and Gly30, residues that match the phospholipid analogue interaction. Hydrophobic contacts can also be compared since the Tyr119 residue next to His120 presented in Figure 2b and Gly30 are included in Rhm with BthTX-II. Rhm has already revealed an antimyotoxic activity against BthTX-II in previous work [5] and could be a potential inhibitor of BthTX-I. The dimeric BthTX-II (tense-state) is necessary for myotoxic activity [8], hence, compounds that change this conformation could decrease its activity. This compound also shows contacts with Gly30 and other important residues, such as Trp31, Tyr120, and the N-terminal region, similar to the amino acids involved in the analogue and myotoxin, BthTX-I. There is no data concerning the inhibitory potential of this compound with CdtsPLA2; however, it has already been shown to decrease the inflammatory cytokines levels and oxidative stress in the mice aortic tissue, besides to inhibit enzymatic, edematogenic, and myotoxic effect of BthTX-II [5,29].

As observed in Rhm, Rhz shows similar interactions with CdtsPLA2. However, it is possible to notice more hydrophobic contact, and contacts with residues are not found in the active site. In addition, Rhz reveals an anchoring in the active site in BthTX-II; in fact, it has already been shown to inhibit the enzymatic activity of this protein [5]. Rhz and Rhm anchor between chains A and B of the BthTX-I and exhibits some important residues in common that were involved in this contact, such as His120, Lys69, His48, Val31, Gly30, and Lys20. Figure 3e,f exhibits that 3MQ also fits in the phospholipid channel, highlighting Lys49, N-terminal residues and His120 of BthTX-I. BthTX-II, besides its myotoxic activity, exhibits low enzymatic activity, and Figure 2b, exhibits the phospholipid analogue in the catalytic site and 3MQ shows to bind in this region. However, it reveals a lower affinity value and fewer interactions. Therefore, this compound has already been shown to poorly inhibit BthTX-II catalytic activity [5]. In a different manner, 3MQ exhibits great interactions in the active site of CdtsPLA2, with the same residues observed in the anchoring between the protein-analogue and protein-commercial inhibitors.

Considering that all these three toxins are from two different species of snake from the Viperidae family, and PLA2 is one of the most abundant components of both Cdt and Bj venoms, it is essential to investigate how distinct compounds can interact with different macromolecules [30,31]. These data indicate that Q, Rhm, 3MQ, and Rhz can anchor in different manners with each toxin. Studies concerning the structural interactions with in vitro analysis, besides the pharmacological assays, could help to better understand diverse mechanisms of inhibition of these compounds. Therefore, our results show that a great candidate to inhibit BthTX-I must interact with residues key-residues cited. BthTX-I docking analysis shows quercetin derivatives could potentially diminish the myotoxic activity by anchoring in the phospholipid hydrophobic channel or interacting with residues next to C-terminal region, which is an essential area to execute the biological effects of this toxin [10]. Nevertheless, it is necessary to perform in vitro and in vivo assays with BthTX-I and CdtsPLA2 to correlate and confirm all the results.

## 4. Materials and Methods

### 4.1. Evaluation of Compounds’ Properties and Preparation

All compounds were analyzed using Molinspiration, SwissAdme and ChEMBL to better understand their characteristics and how they can influence their activities. Physical–chemical properties, the structure and the main biological targets were obtained in ChEMBL. TPSA, lipophilicity, and other features were found in SwissADME and Molinspiration. All molecules were prepared before the molecular anchoring analysis, adding polar hydrogen atoms, and aggregating the Kollman charges and converted to PDBQT.

### 4.2. Proteins Preparation

The PDB (Protein Data Bank—https://www.rcsb.org, accessed on 1 December 2022) was used in this analysis to find the 3D structure of all proteins. Information on the 3D structure of each compound was taken from the PubChem platform (https://puchem.ncbi.nlm.nih.gov, accessed on 1 December 2022). The crystallographic models chosen as the best model for the construction of the theoretical structural models to BthTX-I and BthTX-II from *Bothrops jararacussu* were 3hzd and 2oqd, respectively, and PLA2 from Cdt was 2qog and. First, a general analysis using Swissdock was made between each protein with all compounds to use as a guide for the best regions to interact. Chimera 1.14 program (Ucsf Chimera, 2004) was used to assemble the structural molecular model of the protein and to evaluate the general possibilities of the proteins binding with compounds. After these first steps, proteins were prepared using Autodock Tools, removing the water molecules, besides additional polar hydrogens and aggregating the Kollman charges. Then, the files were converted into PDBQT to perform the calculations of the energy maps (Grid Box) using Autodock Vina [32]. The size was chosen to enclose all amino acids from the catalytic sites or the C-terminal region. The results were obtained using the tools LIGPLOT+ and PyMOL v 2.4 to evaluate the binding energies and orientations of molecules in the microenvironment of the active site of PLA2.

## 5. Conclusions

The results with analogue and the inhibitors corroborate with the literature and indicate the key residues of each toxin to reduce their activity. Q, Rhm, 3MQ, and Rhz showed the highest values of affinity with CdtsPLA2; however, the analysis indicates that 3MQ could better inhibit the enzymatic activity. In BthTX-II, Q anchors in the active site and Rhm in the interface—in vitro and in vivo assays support these results. Rhz also shows great interaction with the active site, and 3MQ revealed less affinity with BthTX-II. Similar results were observed with the compounds and BthTX-I, including affinity values. These docking results emphasize the essential role of Gly30, His48, and Asp49 of BthTX-II and CdtsPLA2 in the interaction with the phospholipids and their hydrolysis. In the case of BthTX-I, the C-terminal region, plus Gly30, His48, Lys49, and His120, are essential to the interaction with the membrane phospholipids and their perturbation and the consequent inflammation induction.

## Figures and Tables

**Table 1:** Physical–chemical characteristics from Q, Rhm, 3MQ and Rhz.

	Q	Rhm	3MQ	Rhz
MW (g/mol)	302.24	316.26	316.26	330.29
Num. rotatable bonds	1	2	2	3
Num. H-bond acceptors	7	7	7	7
Num. H-bond donors	5	4	4	3
TPSA	131.36 Å^2^	120.36 Å^2^	Å^2^	109.36 Å^2^
Lipophilicity (Consensus Log Po/w)	1.23	1.63	1.75	2.02

**Table 2:** Binding free energy (affinity) and rmsd values of each toxin with BPB.

	1º Cluster		2º Cluster	
	Affinity (kcal/mol)	Rmsd (l.b.; u.b.)	Affinity (kcal/mol)	Rmsd (l.b.; u.b.)
BthTX-I:BPB	−5.6	0; 0	−5.4	2.086; 4.796
BthTX-II:BPB	−5	0; 0	−5	0.102; 1.460
CdtsPLA2:BPB	−5.5	0; 0	−5.4	1.129; 2.875

**Table 3:** Binding free energy (affinity) and rmsd values of each toxin with Var.

	1º Cluster		2º Cluster	
	Affinity (kcal/mol)	Rmsd (l.b.; u.b.)	Affinity (kcal/mol)	Rmsd (l.b.; u.b.)
BthTX-I:Var	−8.7	0; 0	−8.5	1.871; 2.797
BthTX-II:Var	−7.1	0; 0	−6.7	2.444; 3.928
CdtsPLA2:Var	−8.3	0; 0	−8.2	1.942; 3.649

**Table 4:** Binding free energy (affinity) and rmsd values of BthTX-I with each quercetin derivative.

	1º Cluster		2º Cluster	
	Affinity (kcal/mol)	Rmsd (l.b.; u.b.)	Affinity (kcal/mol)	Rmsd (l.b.; u.b.)
Q	−8.2	0; 0	−7.9	2.655; 3.657
Rhm	−8.2	0; 0	−8.1	1.625; 2.395
3MQ	−7.9	0; 0	−7.9	2.817; 4.116
Rhz	−8.2	0; 0	−8	2.465; 3.041

**Table 5:** Binding free energy (affinity) and rmsd values of BthTX-II with each quercetin derivative.

	1º Cluster			2º Cluster
	Affinity (kcal/mol)	Rmsd (l.b.; u.b.)	Affinity (kcal/mol)	Rmsd (l.b.; u.b.)
Q	−7.4	0; 0	−7.3	1.688; 2.723
Rhm	−7.7	0; 0	−7.5	0.629; 1.395
3MQ	−6.9	0; 0	−6.7	1.908; 2.325
Rhz	−7.3	0; 0	−6.9	1.826; 6.889

**Table 6:** Binding free energy and rmsd values of Cdt sPLA2 molecular docking with quercetin derivatives.

	1º Cluster		2º Cluster	
	Affinity (kcal/mol)	Rmsd (l.b.; u.b.)	Affinity (kcal/mol)	Rmsd (l.b.; u.b.)
Q	−9.7	0; 0	−8	1.878; 2.823
Rhm	−9.4	0; 0	−8.2	0.530; 1.426
3MQ	−9.4	0; 0	−8.5	1.908; 2.325
Rhz	−8.9	0; 0	−8.8	1.807; 4.896

## Data Availability

Data are contained within the article.
